# ZEB1-activated LINC01123 accelerates the malignancy in lung adenocarcinoma through NOTCH signaling pathway

**DOI:** 10.1038/s41419-020-03166-6

**Published:** 2020-11-15

**Authors:** Miao Zhang, Yaguang Han, Yi Zheng, Yan Zhang, Xin Zhao, Zhenlin Gao, Xinyan Liu

**Affiliations:** 1 Department of Oncology, The NO.1 Hospital of Shijiazhuang in Hebei Province, 050010 Shijiazhuang, Hebei China; 2 Department of Oncology, Hebei Provincial Thoracic Hospital, 050010 Shijiazhuang, Hebei China

**Keywords:** Lung cancer, Cell biology

## Abstract

Growing incidence of lung adenocarcinoma (LUAD) has been detected recently. Multiple long non-coding RNAs (lncRNAs) have been proven as tumor facilitators or inhibitors by extensive works. Present study concentrated on characterizing the potential role of LINC01123 in LUAD. We explored the differential expression of LINC01123 through qRT-PCR and found the amplification of LINC01123 in LUAD cell lines. It was ascertained that LINC01123 was definitely responsible for the malignant processes of LUAD cells. Further, we validated the ceRNA network of LINC01123/miR-449b-5p/NOTCH1 in LUAD via mechanical experiments. As a transcriptional factor related to epithelial mesenchymal transition (EMT), ZEB1 was responsible for the transcriptional activation of both LINC01123 and NOTCH1. The involvement of NOTCH signaling in LUAD was interrogated through evaluating functional changes after treating with FLI-06 (NOTCH pathway suppressor). It showed that FLI-06-caused NOTCH signaling inactivation suppressed malignant functions in LUAD cells. Additionally, LINC01123 facilitated NOTCH1-dependent NOTCH signaling activation. Rescue experiments probed the modulatory function of LINC01123/miR-449b-5p/NOTCH1 in LUAD cellular processes. Altogether, ZEB1-activated LINC01123 accelerates the malignancy in LUAD through miR-449b-5p/NOTCH1 axis-mediated NOTCH signaling pathway, while NOTCH1 boosts ZEB1 in return. These observations suggest the huge potential of LINC01123 as a new target for LUAD therapy.

## Introduction

Lung cancer is a prevalent malignancy that frequently leads to death worldwide^1^. Non-small cell lung cancer (NSCLC) constitutes about 85% of all lung cancer cases^2^. Almost half of NSCLC cases are lung adenocarcinoma (LUAD), the most dominant histological subtype of lung cancer^3^. Despite significant advances in current treatment strategies for LUAD, including surgery, chemotherapy, radiotherapy, and targeted therapy, the prognosis of patients is still unsatisfying over decades^4,5^. Hence, more about LUAD development need to be understood to improve the treatment and prognosis of LUAD patients.

Long non-coding RNAs (lncRNAs) of more than 200 nucleotides are a subtype of regulatory RNA molecules without open reading frame, which limits their capacity in protein-coding^6,7^. Originally, little attention was poured into studies on lncRNAs since they were treated as transcriptional noises. However, it is increasingly demonstrated that specifically expressed lncRNAs are intertwined with biological and pathological reactions, such as differentiation^8^, immune response^9^, metabolism^10^, and tumor exacerbation^11^. As a novel chapter in tumor studies, lncRNAs have been well-addressed to be specifically related to various tumors, among which a set of lncRNAs are proliferative or metastatic markers. For example, lncRNA ZEB2-AS1 speeds up the proliferation of colon cancer cells by miR-143/bcl-2 axis^12^. LncRNA NEAT1 exhibits the proliferation-promoting and metastasis-facilitating functions in breast cancer via regulating carnitine palmitoyltransferase‑1^13^. LncRNA GAS5-AS1 retards cervical cancer growth and metastasis via m^6^A modification of GAS5^14^. Currently, high-throughput analysis including RNA-sequencing and microarray assists the idenfication of lncRNA expression pattern during tumor development^15^. LINC01123 (long-intergenic non-protein-coding RNA 1123) predicts unpleasing survival of patients with head and neck squamous cell carcinoma^16^. However, little about this lncRNA in other cancers like LUAD is known. Not long ago, Hua, Q., et al. found that LINC01123 facilitates cell proliferation in non-small cell lung cancer^17^. Nonetheless, the expression pattern and functional role of LINC01123 in LUAD are still largely unknown.

As the best-known mode of gene expression manipulation, it is hypothesized that lncRNAs possess the miRNA-sponging role in the competing endogenous RNA (ceRNA) mechanism. The specific regulation of microRNAs (miRNAs) on messenger RNAs (mRNAs) could be antagonized by lncRNAs who own similar miRNA recognition elements. A large quantity of reports have reflected the new aspect in post-transcriptional modulation and that the ceRNA role of lncRNAs contributes a lot to the pathogenesis of tumors including LUAD. For instance, lncRNA HCP5 is pro-metastasis in LUAD via the miR-203-sponging role^18^. SNHG6 acts as a ceRNA to sponge miR-26a-5p and promote E2F7 in LUAD^19^.

In this work, we described the facilitating role of LINC01123 in LUAD cell growth, stemness and migration. Besides, the ceRNA mechanism underlying the regulation of LINC01123 on NOTCH1 was probed in LUAD. Meanwhile, the positive regulation of LINC01123 on NOTCH signaling was validated. In addition, the transcriptional activation of LINC01123 and NOTCH1 by ZEB1 was determined in LUAD. Finally, rescue experiments revealed the ZEB1/LINC01123/miR-499b-5p/NOTCH1 axis in LUAD.

## Materials and methods

### Cell lines and reagent

LUAD cell lines (PC9, A549, Calu3, and H1975) and normal human bronchial epithelial cell line BEAS-2B, were both procured from the ATCC (Rockville, Maryland) and allowed to grow in Dulbecco’s modified Eagle medium (Invitrogen, Carlsbad, CA). Cell culture was performed at 37 ^o^C in 95% air and 5% CO_2_, in the presence of medium with 10% fetal bovine serum (FBS) and 1% penicillin–streptomycin (Invitrogen). FLI-06 (NOTCH signaling depressor; 50 μM) was commercially obtained from Med Chem Express USA (Monmouth Junction, NJ).

### RNA isolation and quantitative reverse transcription PCR (qRT-PCR)

The total cellular RNA were isolated from A549 or PC9 cells with the TRIzol reagent (Invitrogen) for reverse transcription using PrimeScript RT reagent Kit (TaKaRa, Shiga, Japan). The cDNA template was subjected to PCR amplification by SYBR Green PCR Kit (TaKaRa). qRT-PCR reaction was run with ABI7500 System (Applied Biosystems, Foster City, CA). Relative expression levels of genes were examined by 2^-ΔΔCt^ method, with GAPDH or U6 taken as the normalization. The sequences of primers were provided in Supplementary Table 1.

### Cell transfection

The short hairpin RNAs (shRNAs) against human LINC01123, NOTCH1, ZEB1 and corresponding negative controls (NC; termed sh-NC), were severally acquired from Genepharma (Shanghai, China). In addition, the pcDNA3.1-LINC01123, pcDNA3.1-NOTCH1 or pcDNA3.1-ZEB1 was, respectively, constructed by inserting indicated cDNA sequence into pcDNA3.1 vectors (Invitrogen). Besides, the miRNAs mimics and NC mimics, miR-449b-5p inhibitor and NC inhibitor, all were obtained from Genechem (Shanghai, China). Cell transfection was processed in A549 or PC9 cells for 48 h by Lipofectamine 2000 (Invitrogen). The transfection efficiency was determined by flow cytometry analysis, and the knockdown or overexpression efficiency was validated via qRT-PCR analysis.

### CCK-8 assay

LUAD cells were placed into 96-well plates (2 × 10^3^/well) with complete culture medium, following the addition of 10 μl of CCK-8 solution for 2 h. Absorbance was evaluated at optical density of 450 nm by applying microplate reader.

### EdU assay

LUAD cells after transfection were seeded into the 96-well plate adding 100 μl of EdU diluent (Ribobio, Guangzhou China). Fixed cells were permeabilized in 0.5% Troxin X-100 and stained with 100 μl of 1 × Apollo^®^ 488, followed by DAPI staining. The proliferative cells were observed under the fluorescent microscope (Leica, Wetzlar, Germany).

### TUNEL assay

Transfected LUAD cells were treated with dUTP-end labeling (Clontech, Mountain View, CA) for TUNEL assay. Following fixation and permeabilization, cells were analyzed by fluorescent microscope after DAPI staining.

### Western blot

The separation of protein samples obtained from LUAD cells was performed on 10% SDS-PAGE, followed by protein transferring onto PVDF membranes (Millipore, Billerica, MA). Subsequent to sealing membranes with 5% nonfat milk, samples were treated with primary antibodies against Bax (ab53154, 1/1000 dilution), Bcl-2 (ab59348, 1/1000 dilution), E-cadherin (ab231303, 1/1000 dilution), N-cadherin (ab18203, 1/1000 dilution), Slug (ab27568, 1/1000 dilution), Vimentin (ab45939, 1/1000 dilution), ZEB1 (ab203829, 1/500 dilution), Snail (ab53519, 1/2000 dilution), Twist (ab49254, 1/400 dilution), NANOG (ab21624, 1/200 dilution), OCT4 (ab19857, 1/1000 dilution), Jag-1 (ab7771, 1/500 dilution), NOTCH1 (ab52627, 1/2000 dilution), Hes1 (ab71559, 1/1000 dilution) and GAPDH (ab181602, 1/10,000 dilution) or Cofilin (ab124979, 1/1000 dilution), as well as the corresponding secondary antibody (ab205718, 1/20,000 dilution). All these antibodies were acquired from Abcam (Cambridge, MA). Membranes were finally analyzed by ECL chemiluminescent detection system (Thermo Fisher Scientific, Rochester, New York).

### Flow cytometry analysis of cell apoptosis

LUAD cells in 6-well plates were washed in cold phosphate buffer saline (PBS) and suspended in 100 μl of binding buffer containing 2.5 μl of FITC-conjugated Annexin V and 1 μl of PI at room temperature for 15 min. Apoptotic cells were collected for flow cytometry analysis (BD Biosciences, Franklin Lakes, NJ).

### Migration assay

Before this experiment, A549 and PC9 cells were starved overnight. Afterwards, cells were collected and re-suspended in serum-free medium, and then planted into the top compartment of 8-um pore chambers inserted in the Transwell apparatus (Corning Incorporated, Corning, NY). Meanwhile, 600 μl of complete culture medium with 10% FBS was added into the bottom compartment. After 24 h of incubation, the cells migrating to the bottom were fixed and stained with 90% methanol and 0.5% crystal violet, respectively. In the end, cell observation was achieved under a microscope.

### Immunofluorescence (IF)

A549 or PC9 cells on coverslips were first fixed and then cultured with the primary antibodies against E-cadherin (ab231303, 1/1000 dilution) and N-cadherin (ab98952, 1/500 dilution) all night, following incubation with FITC-conjugated secondary antibody (ab6785, 1/5000 dilution) for 1 h. All the antibodies were procured from Abcam. Fluorescent staining was analyzed by fluorescence microscope after DAPI staining for visualizing cell nucleus.

### Sphere formation assay

LUAD cells were prepared in the 96-well ultralow attachment plates (Corning; 10/well) in sphere medium for 7 days. At last, the spheres (cell clusters with the diameter >50 mm) were counted.

### Dual-luciferase reporter assays

The reporter vectors pmirGLO-LINC01123-WT/MUT and pmirGLO-NOTCH1-WT/MUT were constructed through inserting full-length LINC01123 or NOTCH1 3’UTR sequences with the wild-type (WT) or mutant (MUT) miR-449b-5p binding sites into pmirGLO vectors (Promega, Madison, WI). Besides, the pGL3-Basis vectors (Promega) were applied to form recombinant vectors covering LINC01123 promoter or NOTCH1 promoter with the WT or MUT ZEB1-binding sites. The co-transfection of above luciferase reporters and indicated transfection plasmids were conducted by using Lipofectamine 2000 (Invitrogen). Forty-eight hours later, cells were collected and the luciferase activity was analyzed via Dual-Luciferase Reporter Assay System (Promega).

### RNA pull-down assay

The extracts from A549 or PC9 cells were prepared for incubation with the biotin-labeled RNA probe (Bio-LINC01123 probe) and Bio-NC probe (probes were constructed by RiboBio), together with the magnetic beads (Invitrogen) at 4 °C for 1 h. The relative enrichments of miRNAs were detected by qRT-PCR in the pull-down complex.

### ChIP assay

ChIP assay was run with the MagnaChIP Kit (Millipore). The crosslinked chromatin was broken into the 200–500-bp fragments and immunoprecipitated with the 2 μg of anti-ZEB1 or 2 μg corresponding anti-IgG all night at 4 °C. Following the addition of magnetic beads, the precipitated chromatin was analyzed via qRT-PCR.

### Nucleus and cytoplasm segmentation

The segmentation of nucleus and cytoplasm in A549 and PC9 cells was conducted as previously suggested^20^.

### Statistical analyses

Each assay included three biological replications. The Graphpad Prism 6 software (GraphPad, San Diego, CA) was applied to process data analysis with Student’s test or one-way analysis of variance followed by post-hoc Tukey’s tests. Results were exhibited with mean ± standard deviation (SD). The probability (*p*) < 0.05 was seen as the cutoff value of statistical significance.

## Results

### LINC01123 enhances the malignant phenotypes of LUAD cells

**Fig. 1:**
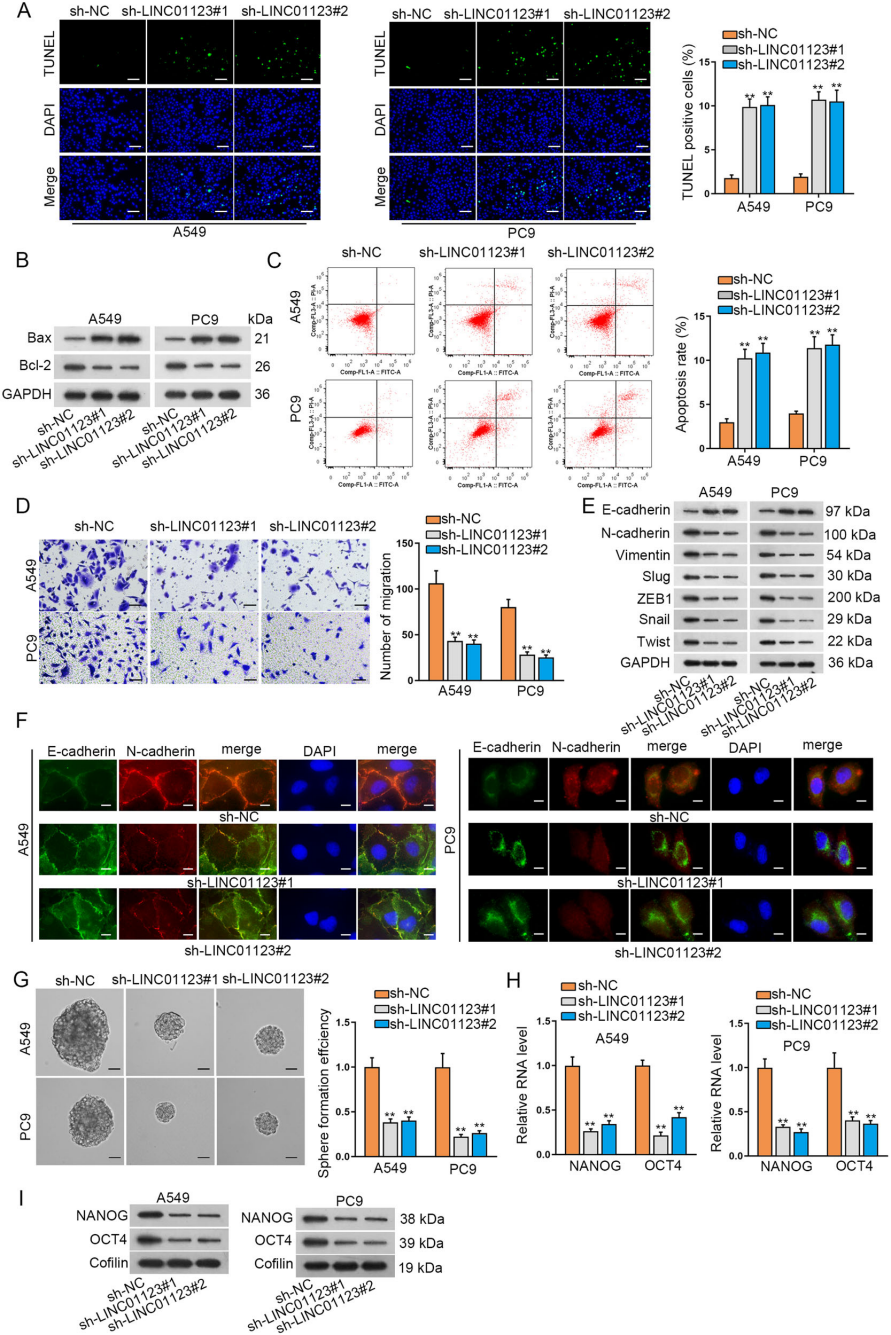
LINC01123 enhanced the malignant behaviors of LUAD cells. **A** TUNEL assay results of LINC01123-depleted LUAD cell apoptosis. **B** Western blot detection of Bcl-2 and BAX protein following LINC01123 depletion. **C** Flow cytometry analysis of LINC01123-depleted LUAD cell apoptosis. **D** Transwell migration assay presented the restrained LUAD cell migration following the ablation of LINC01123. **E** Western blot evaluated the alterations of EMT-related proteins following LINC01123 depletion. **F** IF assay detected the E-cadherin and N-cadherin expression after LINC01123 inhibition. **G** Sphere formation assay assessed the stemness of sh-LINC01123#1/2 transfected LUAD cells and sh-NC transfected cells. **H**, **I** The mRNA and protein levels of NANOG and OCT4 in LINC01123-depleted or non-depleted cells were detected by qRT-PCR and western blot, respectively. **P < 0.01.

As an oncogenic player, LINC01123 is functionally uncharacterized in LUAD. The expression profile of LINC01123 was explored by qRT-PCR. Data described an elevation of LINC01123 in LUAD cell lines (PC9, A549, Calu3, and H1975) compared to control BEAS-2B cells (Supplementary Fig. S1A). To acknowledge the biological function of LINC01123 in LUAD, sh-LINC01123#1/2-mediated knockdown of LINC01123 was conducted in PC9 and A549 cells, which possessed higher endogenous LINC01123 expression (Supplementary Fig. S1B). Then the results of CCK-8 and EdU assays displayed that LINC01123 depletion obviously decreased LUAD cell viability and proliferation (Supplementary Fig. S1C-D). On the contrary, suppression of LINC01123 potently accelerated LUAD cell apoptosis (Fig. 1A). As illuminated by western blot, ablation of LINC01123 led to an accumulation of Bax and a decrease of Bcl-2 (Fig. 1B). Flow cytometry also manifested an augmentation of LUAD apoptosis in the absence of LINC01123 (Fig. 1C).

Thereafter, we also evaluated the impact of LINC01123 on cell migration and epithelial mesenchymal transition (EMT) process in LUAD. It exhibited that inhibition of LINC01123 weakened LUAD cell migration (Fig. 1D). Further, western blot analyzed that the expression of E-cadherin (epithelial marker) was clearly augmented while the levels of mesenchymal markers (including N-cadherin and Vimentin) and EMT-related transcription factors (including Slug, ZEB1, Snail, and Twist) were distinctly reduced under LINC01123 deficiency (Fig. 1E). Consistently, IF assay results suggested that LINC01123 depletion led to enhanced abundance of E-cadherin and reduced signal of N-cadherin (Fig. 1F). In the meantime, we also wondered the influence of LINC0123 on the stemness of LUAD cells. Interestingly, the outcomes of sphere formation assay indicated a reduction of sphere numbers upon LINC01123 knockdown (Fig. 1G). Consistently, the expression of two stemness markers, including NANOG and OCT4, was abated by depleted LINC01123 at both mRNA and protein levels (Fig. 1H–I). Collectively, LINC01123 served a prominent role in aggravating cell proliferation, migration, stemness and EMT in LUAD.

### LINC01123 sponges anti-oncogenic miR-449b-5p

**Fig. 2:**
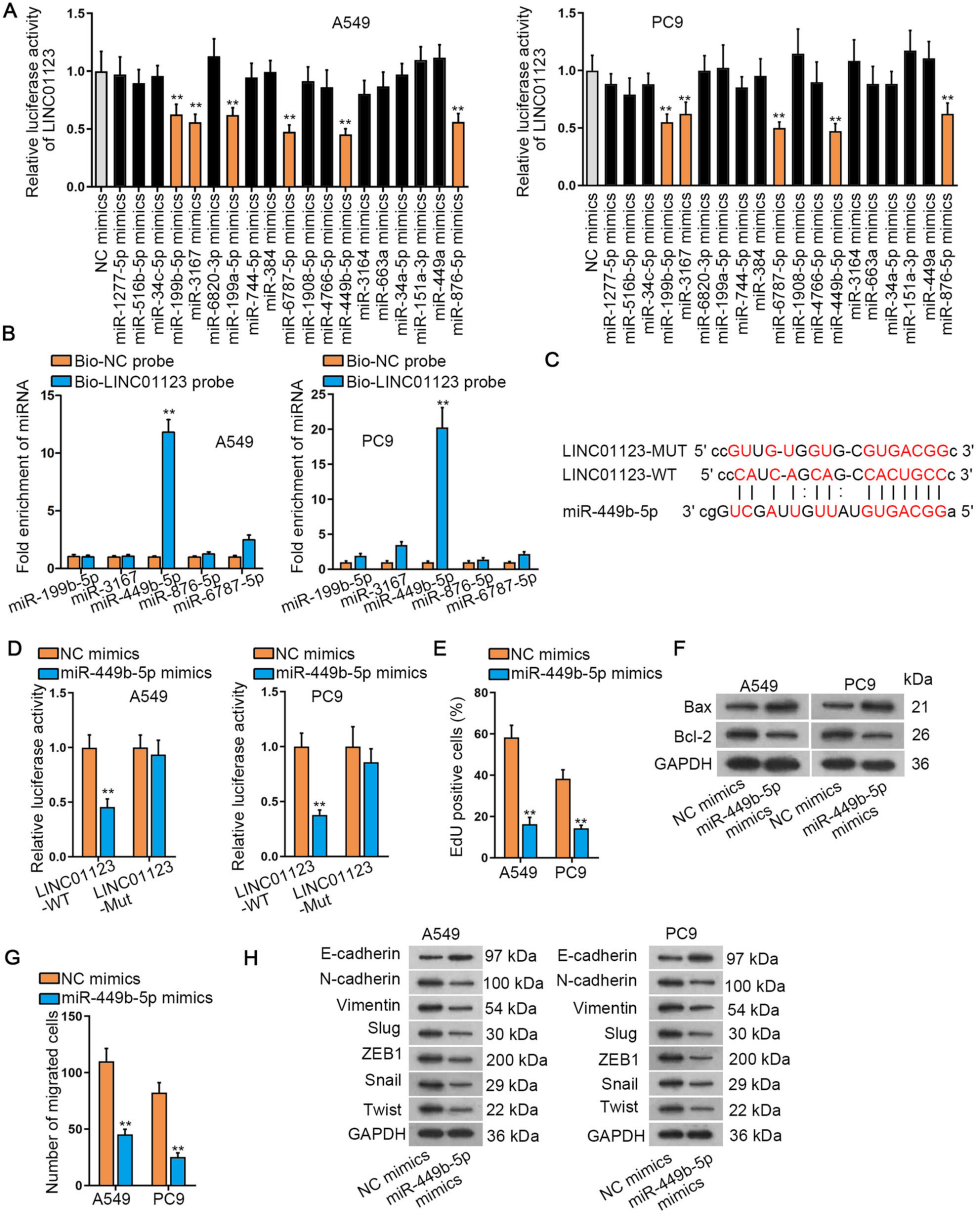
LINC01123 sponged anti-oncogenic miR-449b-5p. **A** LUAD cells were separately transfected with 19 miRNA mimics and luciferase reporters containing wild-type sequences of full-length LINC01123, as indicated by luciferase reporter assay. **B** RNA pull-down assay examined the interaction between LINC01123 and 5 potential miRNAs. **C** Presentation of binding sites between LINC01123 and miR-499b-5p, as predicted by starBase. **D** Luciferase reporter assay conducted in LUAD cells transfected with LINC01123-WT/MUT reporters, NC mimics or miR-499b-5p mimics, as indicated. **E** EdU assay tested LUAD cell proliferation following miR-499b-5p upregulation. **F** Western blot analysis of Bcl-2 and BAX protein levels in LUAD cells transfected with NC mimics or miR-499b-5p mimics. **G** Transwell migration assay detected LUAD cell migration after miR-499b-5p elevation. **H** Western blot analysis of EMT-related proteins in LUAD cells transfected with NC mimics or miR-499b-5p mimics. **P < 0.01.

The regulatory mechanism of LINC01123 in LUAD was explored starting from unveiling the subcellular location of LINC01123. As detected by cell fraction segregation, LINC01123 preferentially distributed in the cytoplasm of LUAD cells (Supplementary Fig. S1E). Based on this, we probed its role as an assumed competing endogenous RNA (ceRNA). As predicted by starBase algorithm (http://starbase.sysu.edu.cn/), LINC01123 harbored binding sequences complementary to the “seed” region of 19 miRNAs. To locate which LINC01123-interacting miRNA was specific in LUAD, we overexpressed 19 miRNAs for subsequent assays (Supplementary Fig. S1F). Luciferase reporter assay results displayed that the luciferase activity of reporters covering LINC01123 was reduced in both A549 and PC9 cells under overexpression of only five miRNAs including miR-199b-5p, miR-3167, miR-449b-5p, miR-876-5p, and miR-6787-5p (Fig. 2A). Afterwards, it demonstrated that biotinylated LINC01123 (Bio-LINC01123 probe) could highly precipitate miR-449b-5p rather than other four miRNAs (Fig. 2B). Bioinformatics data described the binding region between LINC01123 and miR-449b-5p, and we also mutated the miR-449b-5p binding sites in LINC01123 to produce LINC01123-Mut luciferase reporters (Fig. 2C). Subsequently, we observed that the luciferase activity in LUAD cells transfected with LINC01123-WT reporter and miR-449b-5p mimics was reduced while no distinct changes were found in cells transfected with LINC01123-Mut reporter or NC mimics (Fig. 2D). To explore whether miR-449b-5p was involved in LUAD cellular behaviors, we performed functional assays. It was indicated that LUAD cell proliferation was impaired in response to miR-449b-5p mimics (Fig. 2E). By contrast, ectopic expression of miR-449b-5p resulted in augmented apoptosis in LUAD cells, characterized by increased Bax and depleted Bcl-2 (Fig. 2F). Meanwhile, the migratory potential of LUAD cells was retarded by miR-449b-5p mimics (Fig. 2G). With respect to EMT process, western blot pointed that compared with control cells, E-cadherin was enriched whereas the levels of N-cadherin, Slug, Vimentin, ZEB1, Snail and Twist were evidently abrogated in LUAD cells with miR-449b-5p elevation (Fig. 2H). To sum up, LINC01123 sequestered miR-449b-5p, an anti-tumor factor in LUAD.

### LINC01123 upregulates NOTCH1 to activate NOTCH signaling via sequestering miR-449b-5p

**Fig. 3:**
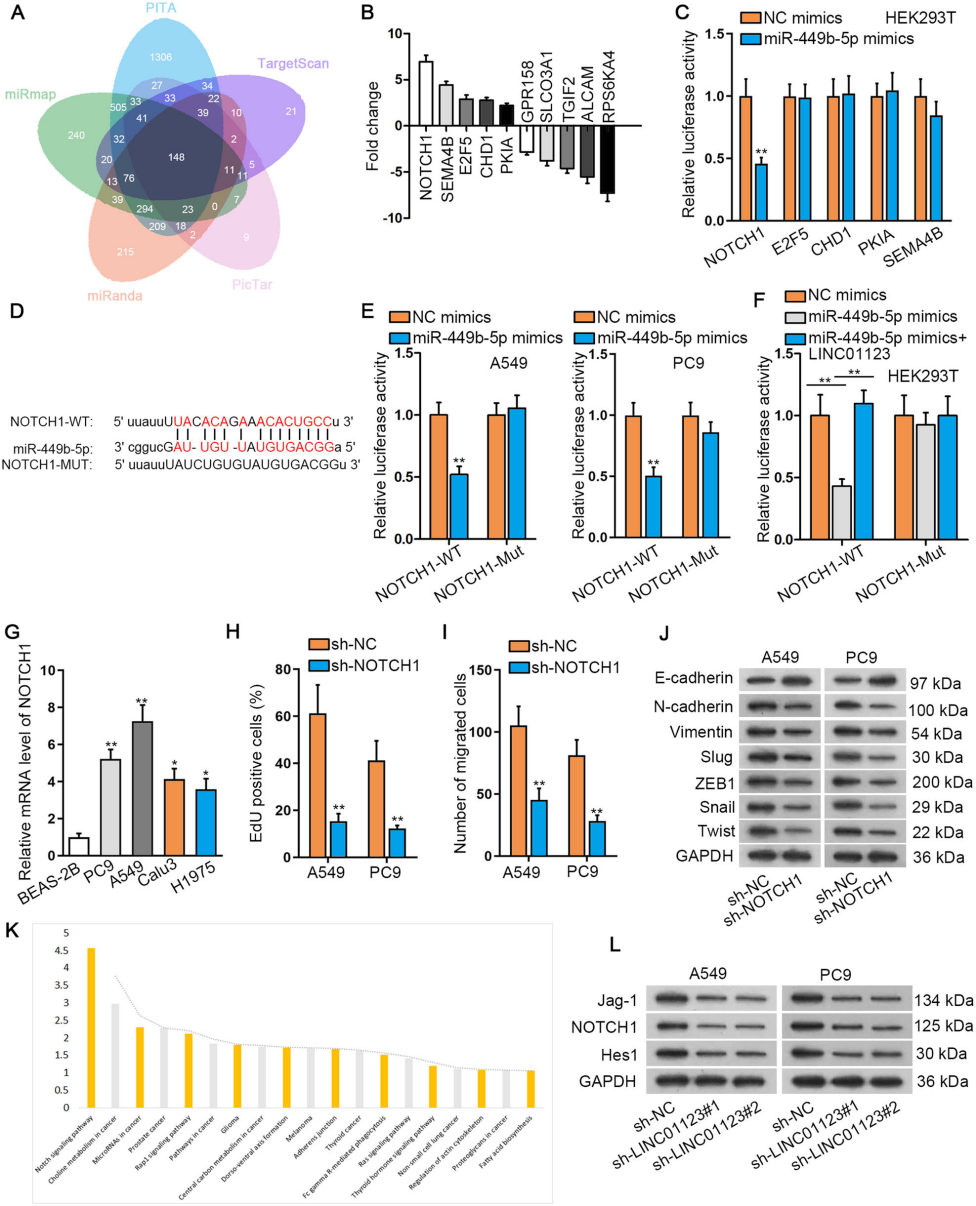
LINC01123 upregulated NOTCH1 to activate NOTCH signaling by sequestering miR-449b-5p. **A** Bioinformatics revelations of miR-449b-5p target genes. **B** Top 10 differentially expressed mRNAs in LUAD cell lines compared with BEAS-2B. **C** Luciferase reporter assay detected the luciferase activity of NOTCH1, E2F5, CHD1, PKIA and SEMA4B responding to NC mimics or miR-449b-5p mimics. **D** Binding sites between NOTCH1 and miR-449b-5p. **E** Luciferase reporter assay examined the luciferase activity of NOTCH1-WT/MUT in response to NC mimics or miR-449b-5p mimics in LUAD cells. **F** Luciferase reporter assay was performed in HEK293T cells transfected with NC mimics, miR-449b-5p mimics or miR-449b-5p mimics+pcDNA-LINC01123; the luciferase activity of NOTCH1-WT/MUT was measured. **G** qRT-PCR revealed NOTCH1 expression in LUAD cell lines and BEAS-2B cells. **H** EdU assay examined LUAD cellular proliferation under the absence of NOTCH1. **I** Transwell migration assay determined LUAD cellular migration following the loss of NOTCH1. **J** Western blot analysis of EMT-associated proteins in face of the absence of NOTCH1. **K** KEGG analysis of the correlation of miR-449b-5p target genes with several pathways. **L** Western blot evaluated changes on proteins related to NOTCH pathway (Jag-1, NOTCH1 and Hes1) following the depletion of LINC01123. **P < 0.01.

A search of miRmap, miRanda, PicTar, TargetScan, and PITA disclosed that 148 mRNAs potentially contained the miR-449b-5p-interacting sites (Fig. 3A). Next, we compared the expression of 148 mRNAs in normal BEAS-2B cells and LUAD cells and unveiled ten significantly altered mRNAs, including top five upregulated and top five downregulated (Fig. 3B). We focused on the five upregulated mRNAs. Notably, we discovered that only the luciferase activity of NOTCH1 3’UTR was apparently inhibited by miR-449b-5p mimics in HEK293T cells (Fig. 3C). Hence, NOTCH1 was chosen as the topic of the following study. The complementary binding sites between miR-449b-5p and NOTCH1 were show in Fig. 3D. As revealed via luciferase reporter assay, miR-449b-5p mimics reduced the luciferase activity of NOTCH1-WT reporter but not that of NOTCH1-MUT reporter (Fig. 3E). Importantly, after LINC01123 was ectopically elevated in LUAD cells (Supplementary Fig. S1G), the reduced luciferase activity of NOTCH1-WT caused by miR-449b-5p mimics was recovered (Fig. 3F). Of note, we discovered that NOTCH1 was highly expressed in LUAD cell lines compared with BEAS-2B cells (Fig. 3G). Afterwards, the functional role of NOTCH1 was examined in LUAD cells. Prior to that, sh-NOTCH1-mediated NOTCH1 suppression was effectively conducted in A549 and PC9 cells (Supplementary Fig. S2A). As a result, NOTCH1 depletion was followed by restrained proliferation, migration and EMT in these two cells (Fig. 3H–J**)**. Moreover, KEGG analysis (https://david.ncifcrf.gov/) suggested that miR-449b-5p target genes were highly correlated with NOTCH signaling pathway (Fig. 3K). Further, depletion of LINC01123 was accompanied by declined protein levels of Jag-1, NOTCH1 and Hes1 (Fig. 3L), hinting a suppressed activity of NOTCH pathway following LINC01123 downregulation. It concluded that LINC01123 upregulated NOTCH1 to activate NOTCH signaling via sponging miR-449b-5p. Afterwards, whether NOTCH signaling pathway could modulate LUAD cellular processes was confirmed utilizing FLI-06 (NOTCH signaling depressor). As presented in Supplementary Fig. S2B, FLI-06 treatment gave rise to retarded NOTCH1 signaling since the levels of Jag-1, NOTCH1, and Hes1 were distinctly declined. Moreover, the results of EdU, TUNEL and western blot analyses manifested that FLI-06 had anti-proliferation and pro-apoptosis effects in LUAD cells (Supplementary Fig. S2C–E). Also, FLI-06 alleviated migration and hindered EMT process in LUAD cells (Supplementary Fig. S2F–G). Likewise, FLI-06 also impaired the stemness of LUAD cells (Supplementary Fig. S2H–I). It was indicative that LINC01123 activates NOTCH signaling to aggravate the malignant progression of LUAD cells.

### MiR-449b-5p/NOTCH1 signaling mediates the function of LINC01123 in LUAD cells

**Fig. 4:**
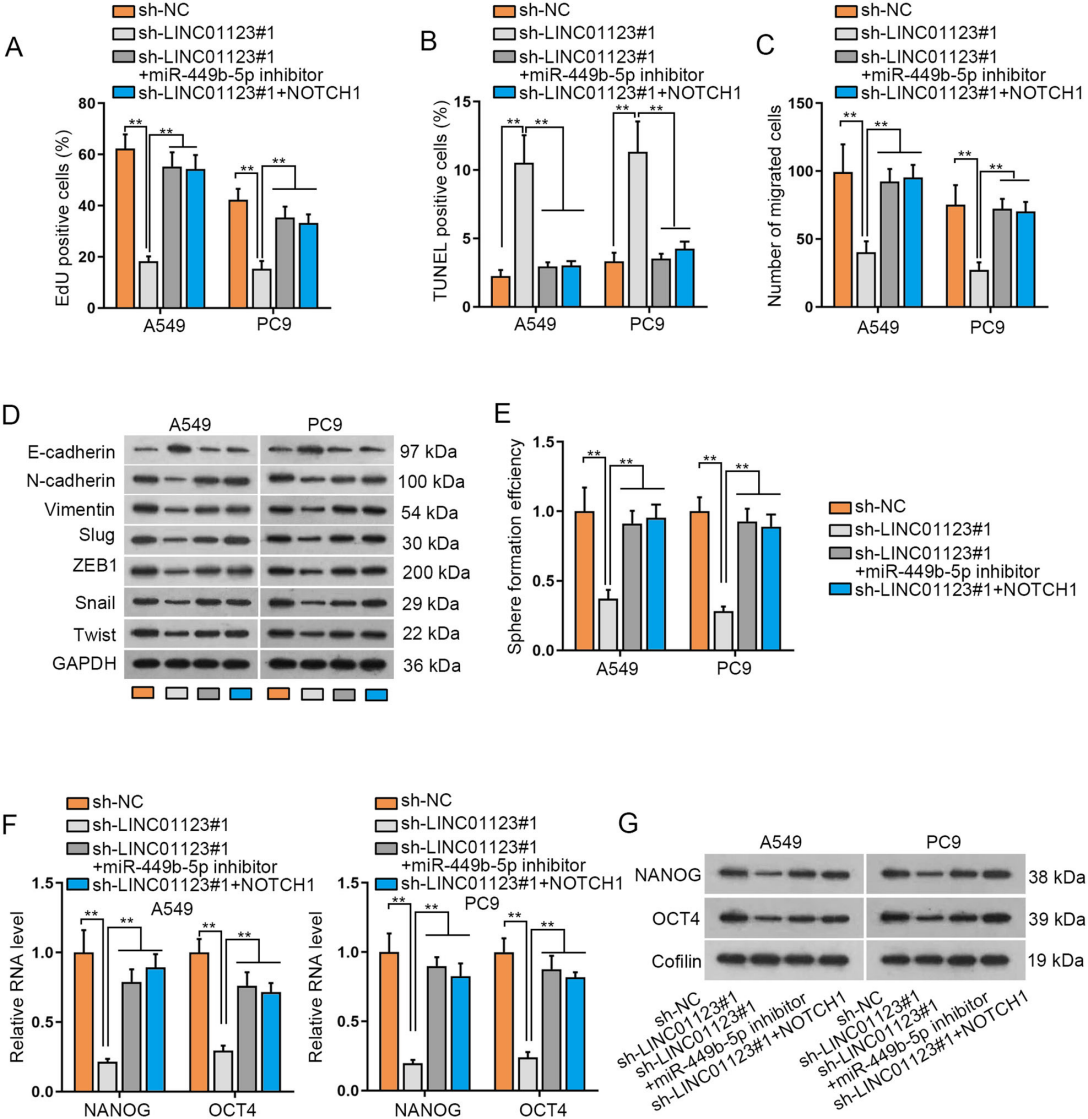
MiR-449b-5p-targeted NOTCH1 mediated the impact of LINC01123 in LUAD cells. **A** EdU assay detected LUAD cellular proliferation under transfection with sh-NC, sh-LINC001123#1, sh-LINC001123#1+miR-449b-5p inhibitor, or sh-LINC001123#1 + NOTCH1. **B** TUNEL assay analyzed the apoptosis of above LUAD cells. **C** Migrated cells were measured following various treatments via Transwell assay. **D** Western blot analyzed EMT-associated proteins in LUAD cells under diverse conditions. **E** Sphere formation ability of indicated LUAD cells was determined by sphere formation assay. **F**, **G** qRT-PCR and western blot assessed the expression of stemness-related genes in indicated LUAD cells. **P < 0.01.

Thereafter, we planned to consolidate the regulatory role of LINC01123/miR-449b-5p/NOTCH1 in LUAD cellular processes. MiR-449b-5p was indeed suppressed by miR-449b-5p inhibitor, which consequently mediated NOTCH1 promotion (Supplementary Fig. S2J). In addition, NOTCH1 was evidently overexpressed after the transfection of pcDNA3.1/NOTCH1 (Supplementary Fig. S2K). Thereafter, we observed that miR-449b-5p depletion could countervail sh-LINC01123#1-caused proliferation impediment, and NOTCH1 promotion mimicked such rescuing effects on LINC01123 depletion-hindered cell proliferation (Fig. 4A). Additionally, the facilitated apoptosis in LINC01123-depleted cells was recovered by either miR-449b-5p inhibition or NOTCH1 augmentation (Fig. 4B). Retarded migration potential of LINC01123-silenced cells was also restored via miR-449b-5p inhibition or NOTCH1 upregulation (Fig. 4C). The effects of LINC01123 suppression on EMT was reversed by NOTCH1 overexpression or miR-449b-5p downregulation (Fig. 4D and Supplementary Fig. S3A). Furthermore, inhibited miR-449b-5p or overexpressed NOTCH1 counteracted the repressed stemness of LINC01123-depleted cells (Fig. 4E). Likewise, miR-449b-5p inhibition or NOTCH1 elevation abated the declined expression of NANOG and OCT4 induced by silenced LINC01123 (Fig. 4F–G and Supplementary Fig. S3B). Taken together, miR-449b-5p and NOTCH1 were required for LINC01123-modulated cellular processes in LUAD.

### LINC01123 and NOTCH1 are both transcriptionally activated by ZEB1 in LUAD

**Fig. 5:**
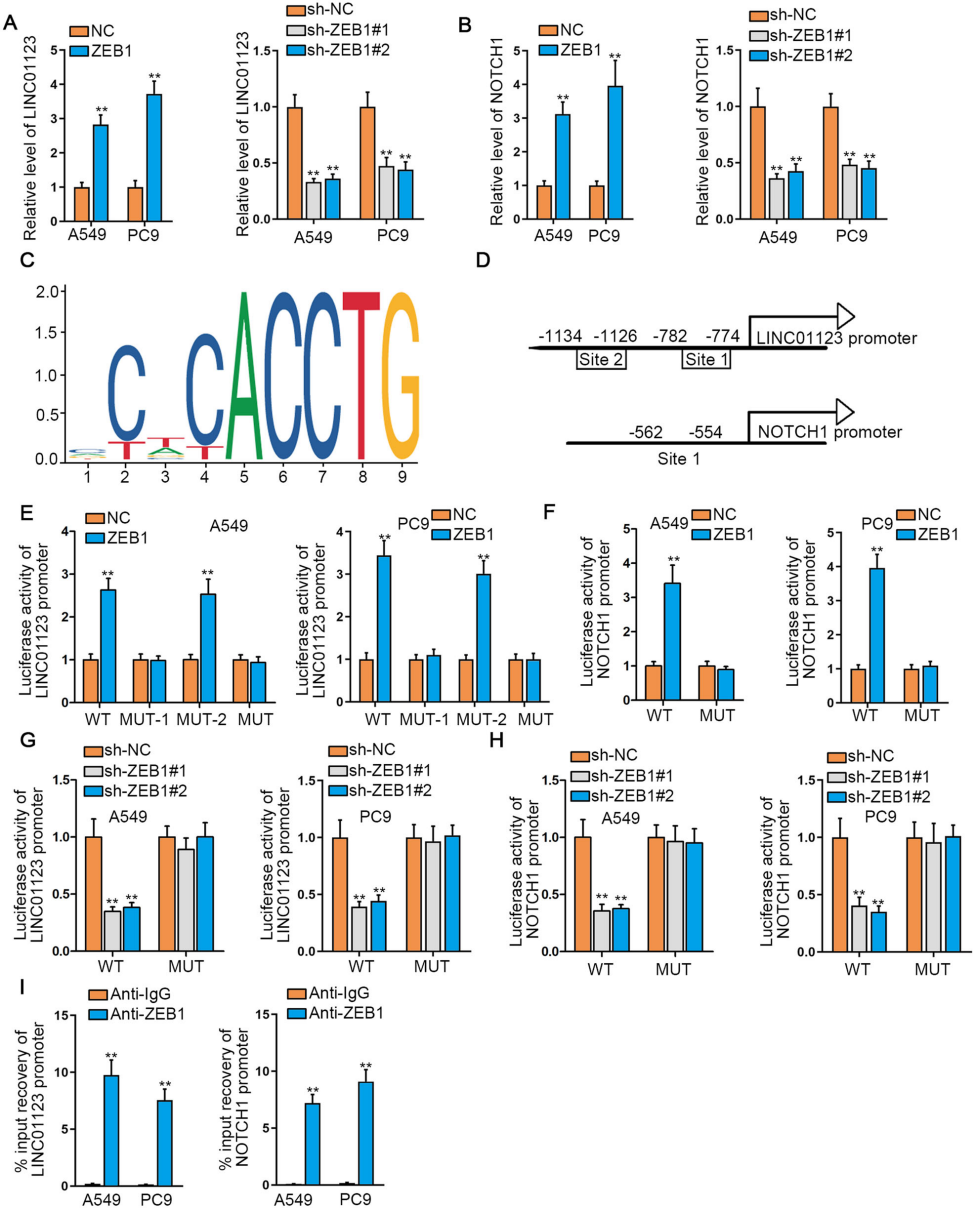
LINC01123 and NOTCH1 were both transcriptionally activated by ZEB1 in LUAD. **A**, **B** qRT-PCR analyzed the expression of LINC01123 and NOTCH1 in LUAD cells responding to ZEB1 upregulation or inhibition. **C** ZEB1 DNA motif from JASPAR algorithm. **D** Schematic presentation of ZEB1-binding sites in LINC01123 promoter and NOTCH1 promoter. **E** Luciferase reporter assays detected the impact of upregulated ZEB1 on the luciferase activity of reporters with different types of LINC01123 promoters (wild, site 1-mutated, site 2-mutated, or site 1 + 2-mutated). **F** Luciferase reporter assays analyzed the influence of upregulated ZEB1 on the activity of wild-type or mutated NOTCH1 promoter. **G**, **H** Luciferase reporter assays analyzed the effect of downregulated ZEB1 on the activity of different types of LINC01123 promoter or NOTCH1 promoter. **I** The occupancy of ZEB1 in LINC01123 promoter and NOTCH1 promoter in LUAD cells was validated by ChIP assay. **P < 0.01.

In view of LINC01123 upregulation and its promotion on EMT, we next explored the transcriptional mechanism upstream of LINC01123. ZEB1 was a pivotal transcription inducer during EMT. We suspected that LINC01123 might be transcriptionally upregulated by ZEB1. Therefore, we ectopically overexpressed or inhibited ZEB1 expression via the transfection of ZEB1 expression vectors or specific targeting shRNAs (Supplementary Fig. S3C). Consequently, we discovered that LINC01123 was positively regulated by ZEB1 (Fig. 5A). Intriguingly, ZEB1 was able to positively regulate NOTCH1 expression (Fig. 5B). Besides, we found that ZEB1 expression was high in LUAD cell lines compared to BEAS-2B controls (Supplementary Fig. S3D), and this trend was consistent with that of LINC01123 and NOTCH1 in LUAD cells. Moreover, it seemed that higher ZEB1 level was accompanied by lower E-cadherin and higher N-cadherin and Vimentin levels (Supplementary Fig. S3E). Thus, we hypothesized that ZEB1 could co-activate LINC01123 and NOTCH1 transcription, so as to affect EMT status in LUAD cells. The ZEB1-binding motif was uncovered (Fig. 5C). The potential binding sites of ZEB1 in LINC01123 promoter and NOTCH1 promoter were indicated in Fig. 5D. The outcomes of luciferase reporter assay elucidated that ZEB1 overexpression led to an increase on the luciferase activity of reporters containing wild-type LINC01123 promoter or the promoter with un-mutated site 1 (only mutated site 2) (Fig. 5E), implying the site 1 in LINC01123 promoter was responsible for the recognition of ZEB1. Similarly, upregulating ZEB1 also led to enhanced luciferase activity of wild-type NOTCH1 promoter (Fig. 5F). Conversely, downregulation of ZEB1 led to opposite results (Fig. 5G–H). Further, ChIP experiments certified the occupancy of ZEB1 in both LINC01123 promoter and NOTCH1 promoter (Fig. 5I). Collectively, ZEB1 was responsible for LINC01123 and NOTCH1 transcriptional activation.

### Ectopic expression of LINC01123 or NOTCH1 reverses the biological behaviors of ZEB1-depleted cells

**Fig. 6:**
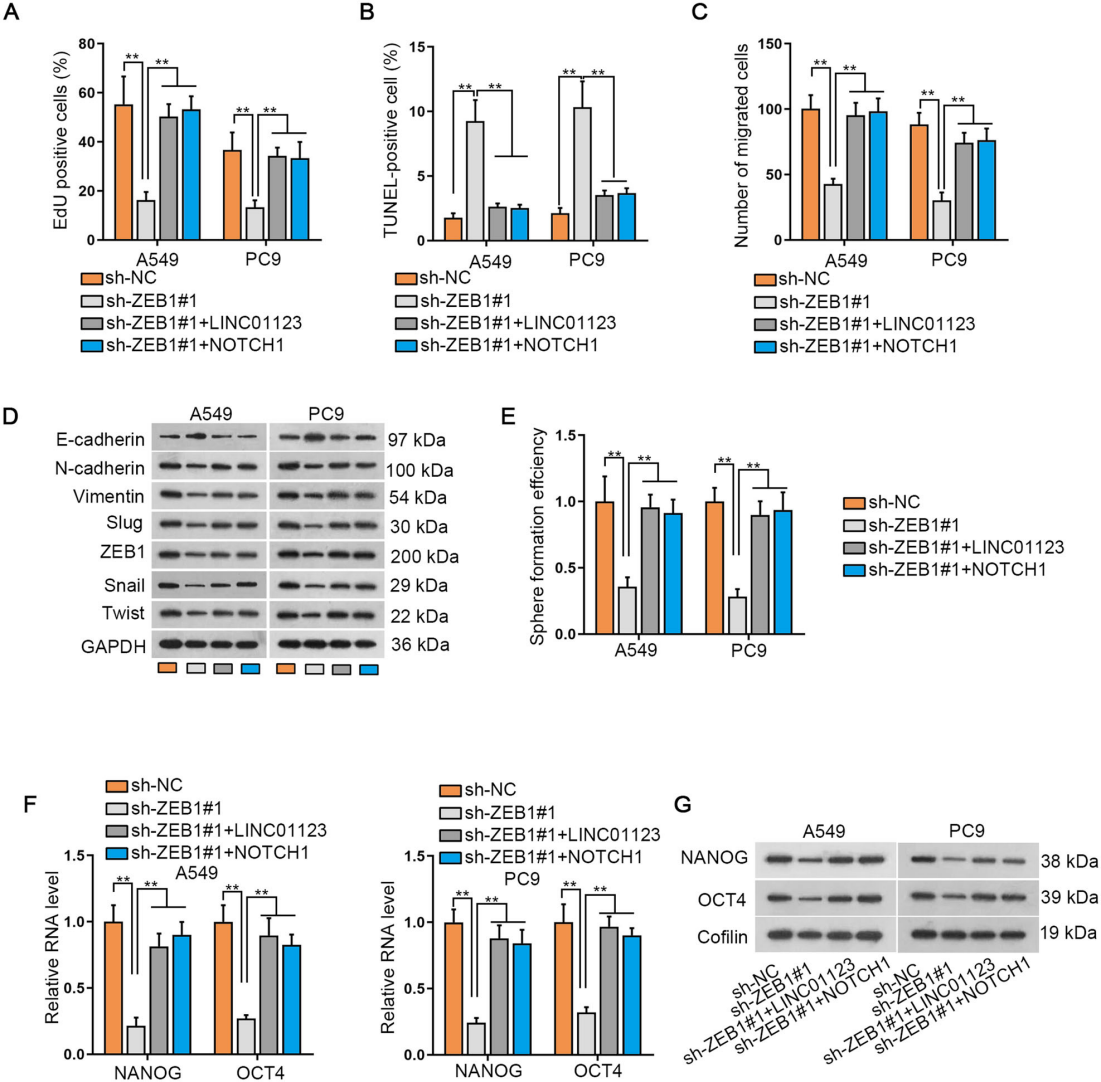
Elevating LINC01123 or NOTCH1 reverted the cellular behaviors of ZEB1-depleted cells. **A**–**G** The proliferation (**A**), apoptosis (**B**), migration (**C**), EMT (**D**), sphere formation (**E**), and stemness (**F**, **G**) of indicated LUAD cells were tested by EdU, TUNEL, transwell and sphere formation assays, and qRT-PCR and western blot analyses, appropriately. **P < 0.01.

Subsequently, ZEB1 was functionally recognized in LUAD cellular processes. As a result, we disclosed that suppressing ZEB1 curbed the proliferation of LUAD cells, while the overexpression of LINC01123 or NOTCH1 could negate this suppression on cell proliferation (Fig. 6A). TUNEL assay results manifested that ablation of ZEB1 gave rise to apoptosis enhancement, whereas LINC01123 or NOTCH1 elevation mitigated this promotion (Fig. 6B). As presented by the results of, transwell migration assay, ZEB1 depletion brought about reduction on cell migration, which was then restored after upregulating LINC01123 or NOTCH1 (Fig. 6C). Concerning EMT process, data described that the influences of ZEB1 depletion on EMT-related proteins were offset by enhanced LINC01123 or NOTCH1 (Fig. 6D and Supplementary Fig. S3F). Moreover, sh-ZEB1#1-caused sphere number reduction could be restored by LINC01123 or NOTCH1 overexpression (Fig. 6E). Furthermore, the it was highlighted that sh-ZEB1#1-caused the decline of NANOG and OCT4 was recovered in response to LINC01123 or NOTCH1 overexpression (Fig. 6F–G and Supplementary Fig. S3G). These findings elucidated that ZEB1 contributes to LUAD progression through LINC01123 and NOTCH1.

## Discussion

Over the last decades, the pivotal impact of lncRNAs on LUAD development has been well-recognized, such as theirregulation on cell metastasis, stemness and growth. A LUAD-specific lncRNA, highly expressed LUADT1 accelerates LUAD cell proliferation through suppressing p27^21^. Qiu et al. presented that CCAT2 serves as a lymph node metastasis biomarker in LUAD^22^. These observations highlight a great chance of breakthrough in unraveling the pathological mechanism in LUAD. Based on the preliminary works, long-intergenic non-protein-coding RNA 1123 (LINC01123) functions as a potent oncogenic player in head and neck squamous cell carcinoma^16^. Noticeably, we addressed that LINC01123 was a powerful factor in promoting LUAD cell growth, migration, EMT and stem-like nature. The facilitating effect of LINC01123 on lung cancer cell proliferation has previously proved by Hua et al.^17^.

In terms of LINC01123-controlled mechanism in LUAD, we discovered the cytoplasmic abundance of LINC01123 in LUAD cells, same as previously revealed^17^. Given the classic role of cytoplasmic lncRNA as a ceRNA, we revealed the potential interplay between LINC01123 and miR-449b-5p. Through the validation of experimental works, it was confirmed that miR-449b-5p was sponged by LINC01123 in LUAD cells. Furthermore, it was disclosed that miR-449b-5p overexpression obviously impaired cell growth, migration and stemness in LUAD. As a consequence of miR-449b-5p elevation, the process of EMT was overtly retarded. However, the pro-apoptosis function of miR-449b-5p was observed in LUAD cells. Previously, extensive studies identified miR-449b-5p as a tumor inhibitor in cancers, including cervical cancer^23^, nasopharyngeal carcinoma^24^, and breast cancer^25,26^. Presently, our work first shed new light on miR-449b-5p function in LUAD together with LINC01123/miR-449b-5p interaction.

NOTCHs, particularly NOTCH1, are well-characterized by their modulatory effects on cellular growth, metastasis, differentiation and apoptosis in human malignancies^27,28^. Furthermore, as reported, several functional lncRNAs exert their vital functions in tumors through Notch signaling. LncRNA FOXD2-AS1 is a tumor driver in colorectal cancer via the regulation of EMT and Notch signaling^29^. LncRNA ZFAS1 serves as an unfavorable prognostic marker and can aggravate glioma progression through activating EMT and Notch signaling pathway^30^. However, the implication of NOTCH signaling in LUAD lacks wide investigation. Herein, we uncovered that NOTCH1 was the downstream target of miR-449b-5p and could be positively mediated by LINC01123. LINC01123 mediated the activation of NOTCH signaling via a NOTCH1-dependent way. Depletion of NOTCH1 led to the impediment of proliferation, migration, EMT and stemness. Results in our study also described the anti-tumor function of NOTCH pathway inhibitor (FLI-06), proving the tumor-facilitating role of NOTCH pathway in LUAD. Additionally, miR-449b-5p inhibition or NOTHC1 upregulation could reverse LINC01123 silence-affected LUAD cellular functions.

ZEB1 is an EMT-related gene that can also serve as a pro-tumourigenic transcription factor. The induction of EMT program by ZEB1 has been addressed in lung cancer^31^. As displayed before, ZEB1 activates the expression of lncRNA ZEB1-AS1 via its direct binding to lncRNA ZEB1-AS1 promoter^32^. Peng et al. suggested that the expression of lncRNA HCCL5 is initiated by ZEB1 in hepatocellular carcinoma^33^. Interestingly, we testified that the transcription activity of NOTCH1 and LINC01123 was related to ZEB1. JASPAR software, ChIP, and luciferase reporter assay displayed that ZEB1 bound to NOTCH1 and LINC01123 promoter regions to initiate their transcription and upregulation. Besides, functional experiments also proved that silencing ZEB1-mediated progression inhibition in LUAD was abolished by LINC01123 or NOTCH1 upregulation.

**Fig. 7:**
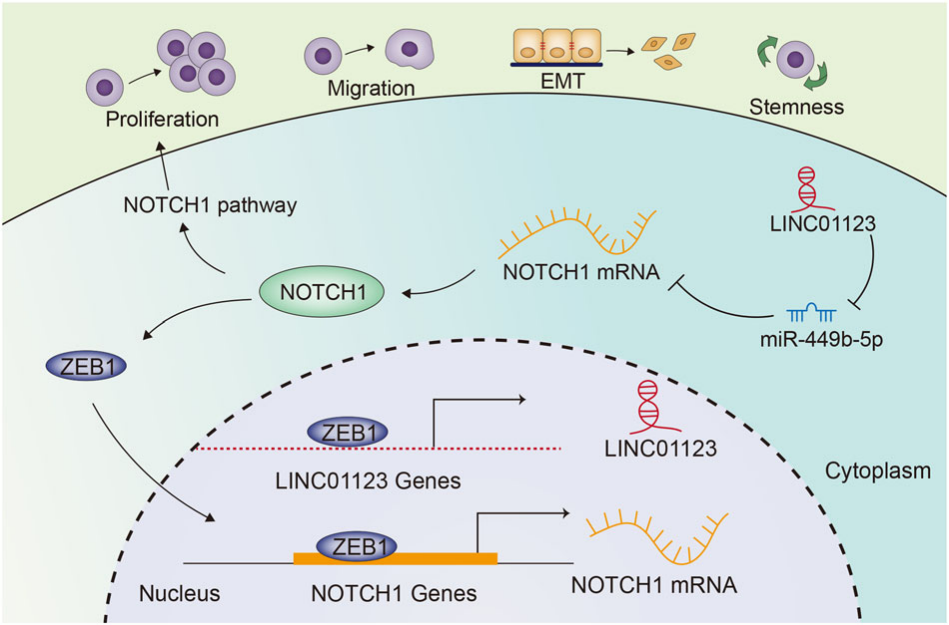
A schematic graph illustrated the mechanism by which LINC01123 promoted lung adenocarcinoma progression and NOTCH pathway activation. Schematic diagram presented that ZEB1-induced LINC01123 mediates LUAD development via miR-449b-5p/NOTCH1 axis-activated NOTCH1 signaling.

Taken together, ZEB1-activated LINC01123 speeds up the malignancy of LUAD through activating NOTCH signaling pathway in a ceRNA manner, while NOTCH1 enhances ZEB1 expression in turn (Fig. 7). Importantly, our findings suggest the huge potential for LINC01123 as a new target for LUAD therapy.

## Supplementary information

Supplementary Figure Legends

Supplementary Figure 1

Supplementary Figure 2

Supplementary Figure 3

Supplementary Table 1
