# Supplementary material for: ZEB1-activated LINC01123 accelerates the malignancy in lung adenocarcinoma through NOTCH signaling pathway
Source: Cell Death Dis. 2020 Nov 15;11(11):981. doi: 10.1038/s41419-020-03166-6 (PMC7667157; doi:10.1038/s41419-020-03166-6)
Supplement: Supplementary file 1 — Supplementary Figure Legends [file 41419_2020_3166_MOESM1_ESM.docx]

**Supplementary Figure 1**

(A) LINC01123 expression profile in LUAD cell lines and BEAS-2B cells was determined by qRT-PCR. (B) LINC01123 knockdown efficacy in A549 and PC9 cells was determined by qRT-PCR. (C) Viability of LINC01123-depleted LUAD cells was tested via CCK-8 assay. (D) Proliferation of LINC01123-depleted LUAD cells was tested by EdU assay. (E) Cytoplasmic location of LINC01123 in LUAD cells determined by nucleus and cytoplasm segmentation. (F) Overexpression efficiency of 19 predicted miRNAs was determined by qRT-PCR. (G) Ectopically elevation of LINC01123 in LUAD cells was confirmed by qRT-PCR. **P < 0.01.

**Supplementary Figure 2**

(A) Knockdown efficacy of NOTCH1 in A549 and PC9 cells was validated by qRT-PCR. (B) Western blot detected the influence of FLI-06 on proteins involved in NOTCH signaling. (C-D) EdU and TUNEL assays evaluated the effects of FLI-06 on LUAD proliferation (C) and apoptosis (D). (E) Apoptosis-associated proteins in LUAD cells with or without FLI-06 were detected by western blot. (F) Transwell assay revealed the impact of FLI-06 on LUAD cell migration. (G) EMT-related proteins in indicated LUAD cells were estimated via western blot. (H) Sphere formation assay examined the sphere formation ability of indicated LUAD cells. (I) The stemness-related proteins were determined via western blot. (J) The expression of miR-449b-5p and NOTCH1 in LUAD cells with or without miR-449b-5p inhibition was assessed by qRT-PCR. (K) NOTCH1 overexpression efficiency was examined through qRT-PCR. **P < 0.01.

**Supplementary Figure 3**

(A) Quantification of western blot bands in Figure 4D. (B) Quantification of western blot bands in Figure 4G. (C) Effective upregulation or inhibition of ZEB1 in LUAD cells was validated by qRT-PCR. (D) The expression pattern of ZEB1 in LUAD cell lines compared to BEAS-2B cells was determined by qRT-PCR. (E) EMT status of LUAD cells and BEAS-2B cells was determined by western blot. (F) Quantification of western blot bands in Figure 6D. (G) Quantification of western blot bands in Figure 6G. *P < 0.05, **P < 0.01.

**Supplementary Table 1**

The sequences of primers used in qPCR and ChIP-qPCR.
