# Supplementary figures and images for: ZEB1-activated LINC01123 accelerates the malignancy in lung adenocarcinoma through NOTCH signaling pathway
Source: Cell Death Dis. 2020 Nov 15;11(11):981. doi: 10.1038/s41419-020-03166-6 (PMC7667157; doi:10.1038/s41419-020-03166-6)

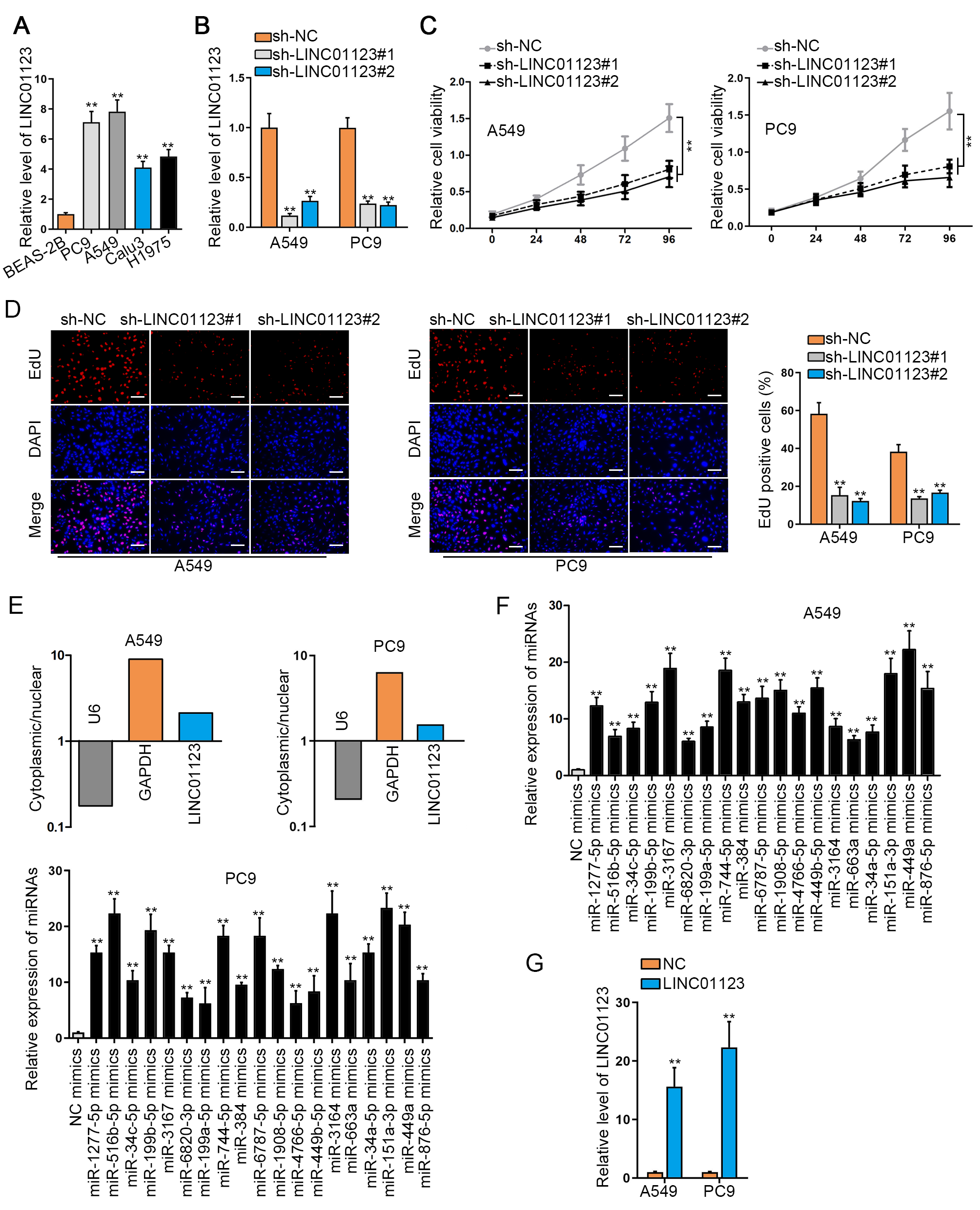

Supplement: Supplementary file 2 — Supplementary Figure 1 [file 41419_2020_3166_MOESM2_ESM.tif]

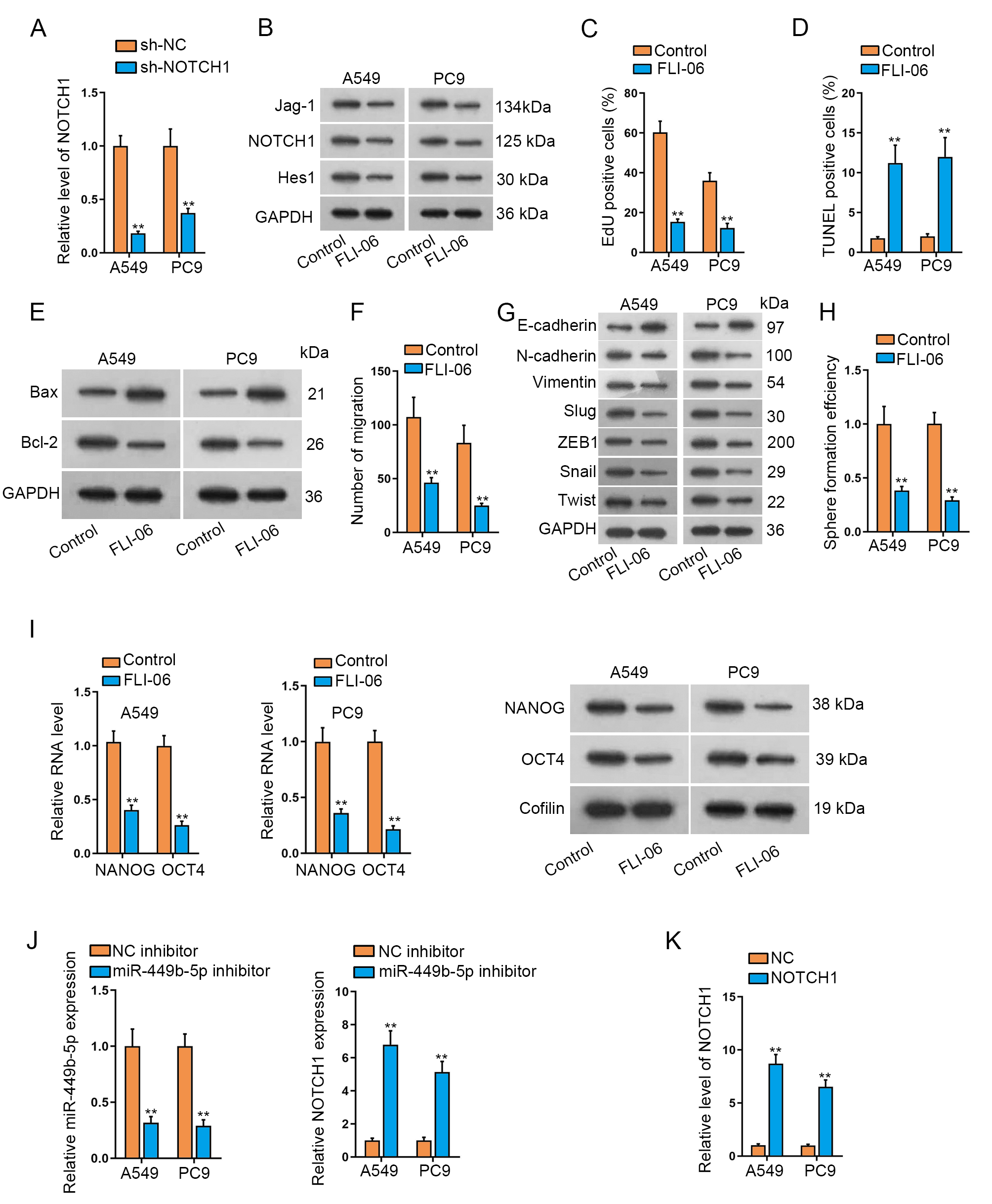

Supplement: Supplementary file 3 — Supplementary Figure 2 [file 41419_2020_3166_MOESM3_ESM.tif]

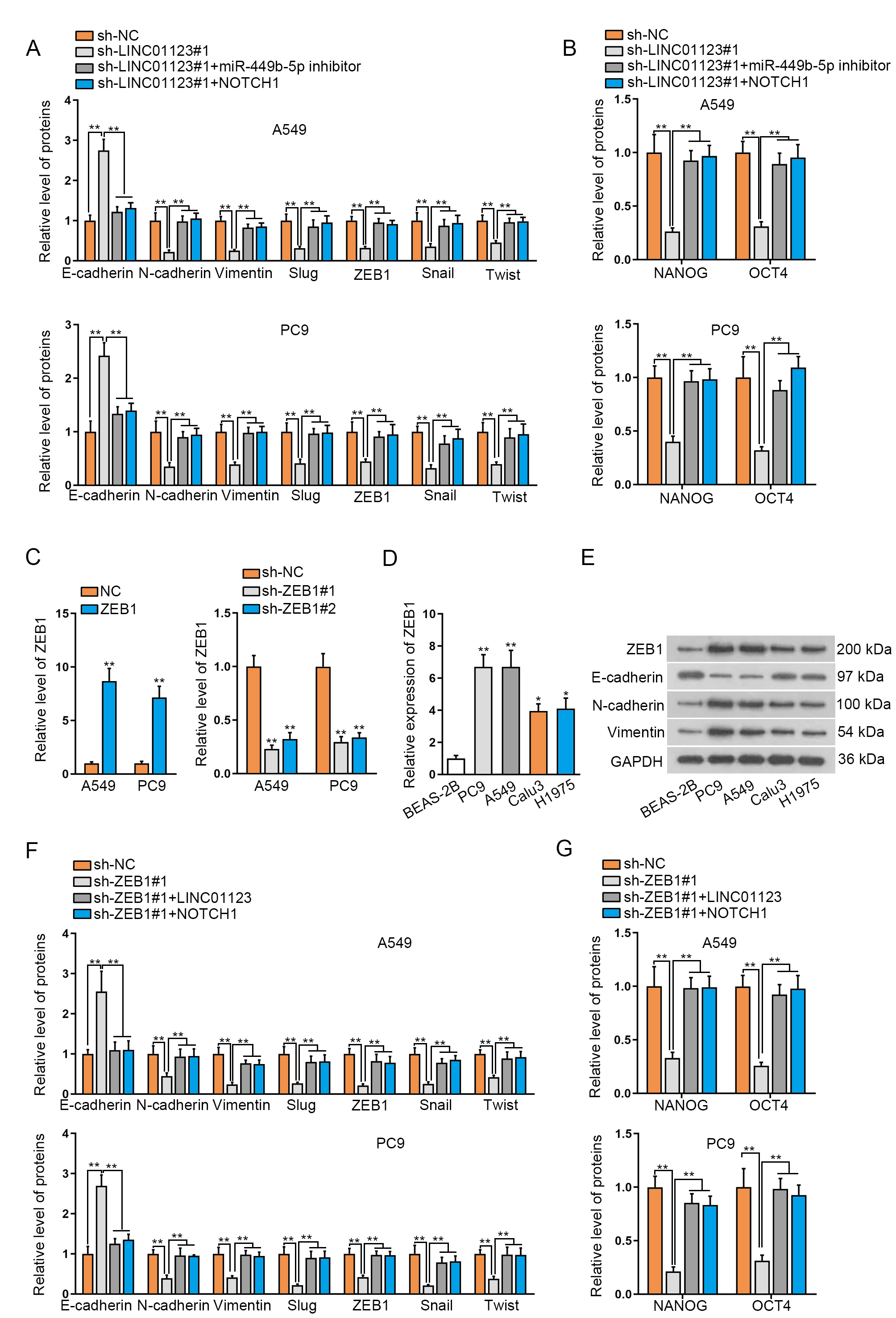

Supplement: Supplementary file 4 — Supplementary Figure 3 [file 41419_2020_3166_MOESM4_ESM.tif]
